# Sex-Related Differences in Proximal Neck Anatomy and Their Consequences in Patients after EVAR: A Matched Cohort Analysis

**DOI:** 10.3390/jcm12154929

**Published:** 2023-07-27

**Authors:** Denise Michelle Danielle Özdemir-van Brunschot, David Holzhey, Spiridon Botsios

**Affiliations:** 1 German Faculty of Health, Witten/Herdecke University, 58455 Witten, Germany; 2 Department of Vascular Surgery and Endovascular Therapy, Augusta Hospital and Catholic Hospital Group, 40472 Düsseldorf, Germany

**Keywords:** abdominal aortic aneurysm, endovascular repair, sex, angulation, endoleak, women

## Abstract

Introduction: Studies comparing male and female patients with abdominal aortic aneurysms have shown that female patients are generally older and more often experience postoperative complications after endovascular and open repair. There are also indications that female patients have more extensive neck pathologies and that they more often have postoperative complications related to proximal neck pathology. Material and methods: This retrospective study describes all consecutive female patients who underwent EVAR between 1 January 2012 and 31 December 2021. Propensity-score matching was used to obtain a matched control male cohort. Propensity scores were generated with the following anatomic parameters: infrarenal and suprarenal angulation, proximal and distal neck diameter and neck length. 1 Female patient was matched with 3 male patients. Results: A total of 160 patients were included, namely 120 male patients and 40 female patients. Due to matching, there were no significant differences regarding infrarenal and suprarenal angulation and proximal and distal neck diameter and length. All-cause and aneurysm-related mortality were comparable (*p* = 0.19 and *p* = 0.98). The necessity of neck-related secondary procedures was significantly higher in female patients (*p* = 0.02). In the multivariate analysis, the female sex was a significant predictor of endoleak type IA within 30 days. However, there was no significant association between intraoperative endoleak type IA and endoleak type IA at the end of follow-up. Conclusions: This study suggests that there was a higher initial incidence of endoleak type IA in female patients, despite thematched preoperative anatomic parameter. Due to the relatively low number of included female patients, conclusions should be drawn carefully.

## 1. Introduction

There are multiple sex-related differences in vascular surgery. For example, in carotid surgery, female patients more often suffer from postoperative stroke in both symptomatic and asymptomatic patients [1]. The male sex is a well-established risk factor for abdominal aortic aneurysm (AAA). Further studies regarding sex differences in patients with abdominal aortic aneurysms (AAA) have shown that female patients are generally older and tend to have longer hospital stays [2,3,4,5]. Moreover, female patients more often experience postoperative complications after endovascular and open aneurysm repair and have higher long-term morbidity and mortality [2,6,7,8,9].

Although the aetiology of the differences in morbidity and mortality are not fully understood, there are a couple of possible explanations: smaller access vessels and higher incidence of undiagnosed cardiovascular diseases, increasing the risk of perioperative cardiovascular events [10]. Another explanation could be differences in the complexity of aneurysm morphology. Every manufacturer of aortic stent grafts has specific requirements concerning the anatomy of the aneurysm, e.g., the length of the neck and angulation. Neck pathology is the most common reason for off-label use [11]. There are indications that female patients more often have extensive neck pathologies [12], which could cause more neck-related complications after endovascular aortic repair (EVAR)*,* e.g., endoleak (EL) type IA [3,13].

In this matched cohort study, we studied the possible consequences of sex differences in patients after EVAR.

## 2. Methods

### 2.1. Patients

In this retrospective study, all consecutive female patients who underwent EVAR between 1 January 2012 and the 31 December 2020 in our centre were included. Asymptomatic, symptomatic and ruptured aneurysms were included. We excluded patients with juxtarenal and suprarenal abdominal aneurysms. Additionally, the chimney technique and fenestrated and/or branched EVAR were excluded from this study. During the study period, EVAR was the first choice of therapy in all patients. The study was approved by the local ethics committee (S-189/2022).

### 2.2. Procedure

All EVAR procedures were performed using commercially available stent grafts, including the Talent (Medtronic, Santa Rosa, CA, USA), Endurant II and Endurant IIS (Medtronic, Santa Rosa, CA, USA), Excluder (W.L. Gore and Associates, Flagstaff, AZ, USA), Zenith (Cook, Bloomington, IN, USA), Powerlink (Endologix, Irvine, CA, USA), Ovation (Endologix, Irvine, CA, USA), E-tegra (Jotec, Hechingen, Germany) and INCRAFT (Cordis Corp, Bridgewater, NJ, USA). All procedures were performed by a vascular surgeon, and in all procedures, general anaesthesia was used. Both surgical exposition and total percutaneous procedure were used to gain access to the common femoral arteries. During the first few study years, surgical exposition was performed via a small incision in the groin, exposing the common femoral artery. During the latter study years, total percutaneous procedure was preferred.

After angiography, the lowest renal artery was marked and the stent graft was deployed. The contralateral leg was cannulated and both limbs were deployed after marking both internal iliac arteries. If an EL1A was seen during the intraoperative angiography, a balloon moulding was performed. If possible, a persistent EL1A was treated with a Palmaz stent, Aptus stapler^®^ or cuff implantation. When both procedures were not successful or not possible, a secondary procedure, e.g., the chimney technique, was planned.

### 2.3. Image Analysis

The maximal diameter of the aneurysm was derived from CT angiography and was based on outer wall to outer wall measurement perpendicular to aorta in an anterior–posterior plane, as advised in international guidelines [14]. EL was defined as extravasation of contrast material between the prosthesis and the aneurysm wall, and EL1A was defined as the extravasation of contrast material at the proximal sealing zone. Technical success was defined as the successful introduction and deployment of the device without surgical conversion, mortality, EL1 or EL3 or limb obstructions, whereas clinical success was defined as the successful deployment of the device without aneurysm-related mortality, EL1 or EL3, graft infection, aneurysm expansion of ≤5 mm during follow-up, aneurysm rupture or conversion to open surgery during the follow-up period [14].

### 2.4. Follow-Up Protocol

After the procedure, patients were observed for 24 h at our intensive care ward. If glomerular filtration rate was >30 mL/min, a computed tomography angiography (CT-angiography) was performed during the hospital stay. Duplex sonography was performed at 6 months, 12 months and yearly thereafter. In the case of the enlargement of the aneurysm sac or insufficient visibility, a CT-angiography was performed. In case of EL1 or EL3, an adjunctive procedure, e.g., relining, cuff implantation, the chimney technique, extension with an iliac side branch or conversion to open surgery, was advised.

### 2.5. Statistical Analysis

SPSS version 28 (IBM Corp., Armonk, NY, USA) was used for all statistical analyses. Propensity-score matching was used to obtain a matched control cohort. Propensity scores were generated using the following anatomic parameters: infrarenal and suprarenal angulation, proximal and distal neck diameter and neck length. We choose to match 3 male patients with 1 female patient.

Categorical data were presented as absolute numbers and percentages; they were compared using Pearson’s X^2^ test (and Fisher’s exact test when *n* < 5), whereas continuous variables were presented as means with standard deviation (SD) and were compared using Student’s *t*-test. The Kaplan–Meier method with a log-rank test was used to assess all-cause, aneurysm-related mortality and the use of secondary neck-related procedures. We considered *p*-values of <0.05 as significant. Binary logistic regression analysis was performed. All possible confounding factors were included in the binary logistic regression analysis.

## 3. Results

A total of 120 patients were included in the study: 120 male and 40 female patients. Although we matched patients in terms of the abovementioned anatomic parameters, there were significant differences regarding mean age (74.3 years ± 7.7 for male and 76.8 years ± 6.9 for female patients, *p* = 0.04) and mean aneurysm diameter (53.8 mm ± 13.2 for male and 49.7 mm ± 10.7 for female patients, *p* = 0.04), as seen in Table 1 and Table 2. There were no significant differences regarding cardiovascular comorbidities and other aneurysm characteristics. There were two male patients with ruptured aneurysms, while none of the female patients presented with a ruptured aneurysm (*p* = 0.41).

There were no significant differences when comparing the incidence of intra-operative adjunctive procedures (26.7% versus 30.0%) and intra-operative neck-related adjunctive procedures (7.5% versus 3.3%). However, female patients more often had an intra-operative EL1A (3.3% versus 12.5%), as seen in Table 3. Intra-operative neck-related adjunctive procedures included the implantation of a cuff (five male patients, two female patients), Palmaz^®^ stent (two male patients, two female patients) or the use of the Aptus^®^ EndoStapler (three male patients, one female patient). In one male patient, cuff implantation was combined with Aptus^®^ Endostaplers. For female patients, technical success was 77.5%; for male patients, technical success was 91.7%. Reasons for the failure of technical success in women in included an occluded limb (*n* = 4) and EL1A (*n* = 5).

Nine patients (four male and five female patients) left the operation room with EL1A. In three of the four male patients, EL1A was confirmed in postoperative CT angiography. One male patient refused further treatment. The other two patients were treated with a Palmaz^®^ stent during initial hospitalisation. Regarding the female patients with EL1A, there were five female patients who left the operation room with an EL1A, and EL1A was confirmed in all female patients at postoperative CT angiography. There was one additional female patient with EL1A at the postoperative CT angiography. Treatment was advised in all female patients. However, one female patient refused treatment. Two female patients were treated with a Palmaz^®^ stent and another with the EndoStapler^®^. For the remaining two female patients, treatment was planned at a later stage.

Thirty-day mortality was 3.3% for male patients, Table 4. One male patient died intraoperatively; this patient was admitted because of a ruptured aneurysm. Another male patient died because of postoperative intestinal ischemia. The other two male patients died because of cardiopulmonary comorbidity.

At the maximal follow-up period, female patients had to undergo neck-related adjunctive procedures more often (6.7% versus 17.5%, *p* = 0.04), and there was a trend towards a higher incidence of EL1A (9.2% for male patients and 20.0 for female patients, *p* = 0.07).

Further multivariate analyses revealed that female sex was an independent predictor of EL1A, both within 30 days and at maximal follow-up, as shown in Table 5. Infrarenal angulation was a significant predictor for intra-operative EL1A; however, this was not the case thereafter.

**Figure 1:**
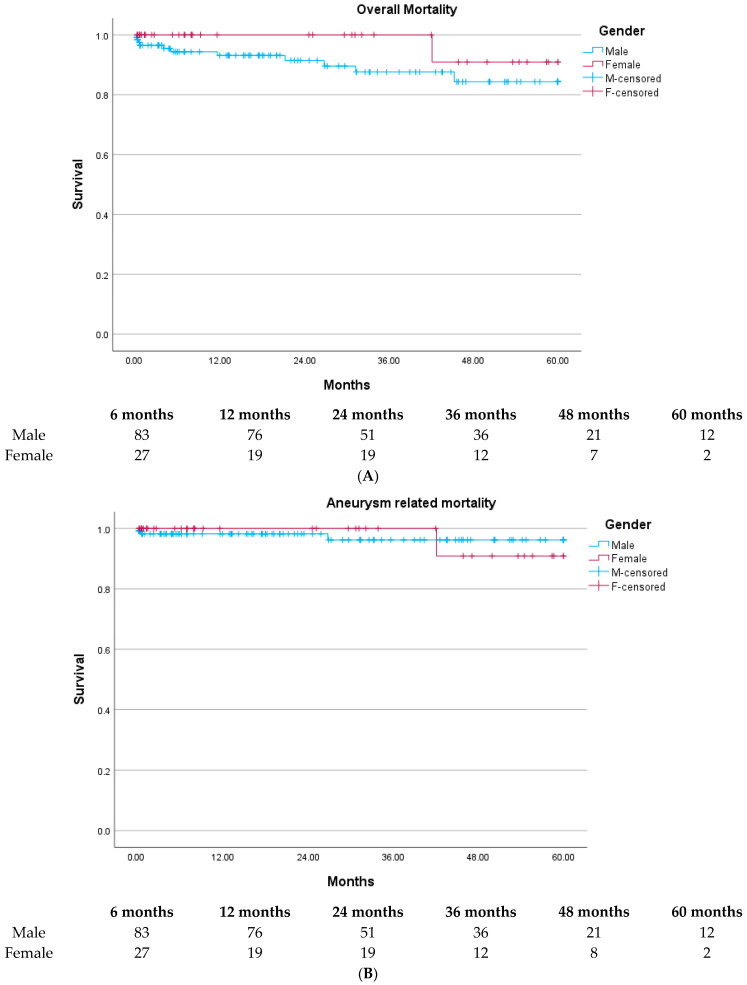

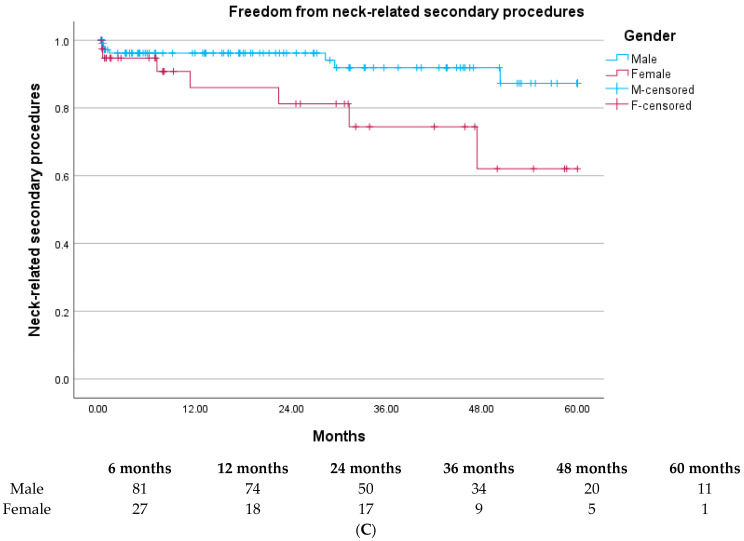
(**A**): All-cause mortality (log rank 1.74, *p* = 0.19). (**B**): Aneurysm-related mortality (log rank 0.02, *p* = 0.98). (**C**): Freedom from neck-related secondary procedures (log rank 5.34 *p =* 0.02).

All-cause and aneurysm-related mortality was comparable in both groups (*p* = 0.19 and *p* = 0.98), as seen in Figure 1A,B. The need for neck-related secondary procedures was significantly higher in female patients (*p* = 0.02), Figure 1C.

## 4. Discussion

There are multiple differences when comparing female and male patients with an AAA. The exact mechanism for this difference is unknown, but sex hormones probably play an important role. Oestrogen seems to reduce macrophage matrix metallopeptidase-9 production and therefore reduce the degradation of the extracellular matrix [15,16,17]. Other studies described a reduction in inflammatory response after rising serum oestrogen levels [17]. Another difference between male and female patients is the higher risk of rupture at any given aneurysm diameter in women [18]. The reason for this is probably the generally smaller body size of women, and there are indications that aortic size index should be used in female patients instead of aneurysm diameter [19,20].

There are also multiple studies that describe a higher morbidity and mortality in female patients after EVAR [8,21,22,23,24,25]. There are a couple of possible explanations for these differences. First, it seems that the medical treatment of cardiovascular risk factors is insufficient in female patients [23,26]. Moreover, the diagnosis of cardiovascular comorbidities, such as coronary artery disease, is delayed in female patients, leading to a more serious clinical situation. Female patients tend to be older than male patients during intervention. This finding was confirmed in this study. Another possible explanation could be anatomical differences in male and female patients. Previous studies have reported on the anatomical differences between female and male patients with an AAA [3,11,12,27]. Female patients present with more angulated aneurysms and shorter neck lengths.

In this study, we used a matched control group to adjust for the anatomical differences between male and female patients. Nonetheless, there a necessity to perform more neck-related secondary procedures in female patients (*p* = 0.04), and there was a tendency towards a higher incidence of EL1A in female patients at the end of the follow-up period (*p* = 0.07). In the multivariate analysis, female sex was a significant predictor of EL1A at 30 days and at the end of the follow-up period. A possible explanation for this finding in this study could be the fact that the mean proximal oversizing was more pronounced in the male population (30.1% versus 24.8%), although the oversizing of 24.8% is within the margins of the instructions for use. Another explanation could be that there is a combination of anatomical factors in female patients, e.g., neck length and infrarenal angulation, which increase the risk of EL1A.

Despite the higher incidence of EL1A and the necessity to perform neck-related secondary procedures, there were no differences regarding all-cause and aneurysm-related mortality.

### Limitations

The main limitation of this study is its retrospective nature with its associated biases. Additionally, as AAA mainly presents in male patients, the number of female patients is limited, despite a study period of 9 years. To overcome this, a multicentre study could be performed. Another possible cause of bias is the large number of different manufacturers used in this study. It should be stated, however, that in the majority of the patients, an Endurant II or IIS stent graft was used.

Another limitation is the large portion of patients lost to follow-up. Despite a long study period, mean follow-up duration was 25.6 months.

## 5. Conclusions

This study suggests a higher incidence of EL1A in female patients, despite matched preoperative anatomic parameters. Possible explanations could be the higher degrees of oversizing in the male population and the possibility that female patients more often have a combination of hostile neck features. These outcomes should be kept in mind when EVAR is planned in female patients. More studies are necessary to confirm our findings and to understand the mechanisms causing these sex-related differences.

## Figures and Tables

**Table 1:** Patient characteristics.

	Male (*n* = 120)	Female (*n* = 40)	*p*-Value
Age	74.3 ± 7.7	76.8 ± 6.9	0.04
Cardiovascular risk factors			
Hyperlipidaemia	53 (44.2%)	17 (14.2%)	0.82
Current or former smoker	50 (41.7%)	20 (16.7%)	0.38
Hypertension	91 (75.8%)	32 (26.7%)	0.64
DM	18 (15.0%)	6 (15.0%)	0.99
Cardiovascular comorbidities			
CAD	49 (40.8%)	12 (30.0%)	0.21
CVD	12 (10.0%)	5 (12.5%)	0.67
PAD	12 (10.0%)	5 (12.5%)	0.67
COPD	20 (16.7%)	12 (30.0%)	0.07
CKD	4 (3.3%)	2 (5.0%)	0.64
Dialysis	2 (1.7%)	1 (2.5%)	0.74
Rupture	2 (1.7%)	0 (0.0%)	0.41

CAD = coronary artery disease; CKD = chronic kidney disease; COPD = chronic obstructive pulmonary disease; CVD = cerebrovascular disease; DM = diabetes mellitus; PAD = peripheral artery disease.

**Table 2:** Aneurysm characteristics.

	Male (*n* = 120)	Female (*n* = 40)	*p*-Value
Mean aneurysm diameter (mm)	53.8 ± 13.2	49.7 ± 10.7	0.04
Infrarenal angulation (°)	36.0 ± 19.5	40.2 ± 29.8	0.15
Suprarenal angulation (°)	22.5 ± 18.5	24.3 ± 23.7	0.31
Proximal neck diameter (mm)	21.6 ± 2.2	21.4 ± 4.2	0.32
Distal neck diameter (mm)	22.5 ± 3.1	22.4 ± 5.2	0.43
Neck length (mm)	21.8 ± 12.9	18.6 ± 13.7	0.09
Thrombus neck	16 (13.3%)	9 (22.5%)	0.18
Conical neck	7 (5.8%)	3 (7.5%)	0.73
Calcification neck	3 (2.5%)	3 (7.5%)	0.16

**Table 3:** Intra-operative parameters.

	Male (*n* = 120)	Female (*n* = 40)	*p*-Value
Mono-iliac stent	3 (2.5%)	2 (5.0%)	0.43
Mean proximal oversizing	30.1 ± 15.4	24.8 ± 16.7	0.06
Adjunctive procedures	32 (26.7%)	12 (30.0%)	0.68
Neck-related adjunctive procedures	9 * (7.5%)	4 (3.3%)	0.62
Cuff implantation	5 (4.2%)	2 (5.0%)	0.82
Palmaz^®^ stent	2 (1.7%)	2 (5.0%)	0.24
Aptus ^®^ EndoStapler	3 (2.5%)	1 (2.5%)	1.00
Intra-operative EL1A (end of the procedure)	4 (3.3%)	5 (12.5%)	0.03
Conversion in laparotomy	1 (0.8%)	0 (0.0%)	0.56
Technical success	110 (91.7%)	31 (77.5%)	0.02
Manufacturer			
Endurant II (Medtronic)	81 (67.5%)	26 (65.0%)	0.77
INCRAFT (Cordis)	23 (19.2%)	5 (12.5%)	0.34
E-tegra (Jotec)	3 (2.5%)	0 (0.0%)	0.31
Zenith (COOK)	2 (1.7%)	2 (5.0%)	0.24
Ovation (Trivascular)	4 (3.3%)	4 (10.0%)	0.09
Other	7 (5.8%)	3 (7.5%)	0.71

EL = endoleak. * in one male patient cuff implantation was combined with Aptus^®^ Endostaplers.

**Table 4:** Postoperative parameters.

	Male (*n* = 120)	Female (*n* = 40)	*p*-Value
Thirty-day outcome			
Mortality	4 (3.3%)	0 (0.0%)	0.24
Secondary procedures	14 (11.7%)	9 (22.5%)	0.09
Neck-related secondary procedures	2 (1.7%)	3 (7.5%)	0.07
Cardiac complications	1 (0.8%)	0 (0.0%)	0.56
Pulmonary complications	4 (3.3%)	1 (2.5%)	0.79
EL1A	3 (2.5%)	6 (15.0%)	<0.01
Follow-up			
Clinical success	97 (80.8%)	28 (70.0%)	0.15
Mean shrinkage of aneurysm sac	1.7 ± 7.9	−2.2 ± 5.9	<0.01
Rupture	1 (0.8%)	1 (2.5%)	0.41
Secondary procedures	32 (26.7%)	15 (37.5%)	0.19
Neck-related secondary procedures	8 (6.7%)	7 (17.5%)	0.04
EL1A	11 (9.2%)	8 (20.0%)	0.07

EL = endoleak.

**Table 5:** Multivariate analysis.

	OR (95% CI)	*p*-Value
Intra-operative EL1A		
Sex	1.00 (0.08–1.71)	0.20
Age	1.01 (0.94–1.18)	0.33
Infrarenal angulation	1.04 (1.00–1.07)	0.03
Suprarenal angulation	1.00 (0.95–1.03)	0.56
Proximal neck diameter (mm)	1.26 (0.90–1.77)	0.18
Distal neck diameter (mm)	0.96 (0.73–1.26)	0.75
Neck length (mm)	0.99 (0.94–1.05)	0.72
EL1A at 30 days		
Sex	0.20 (0.04–0.89)	0.03
Age	1.08 (0.97–1.20)	0.17
Infrarenal angulation	1.02 (0.99–1.05)	0.22
Suprarenal angulation	0.99 (0.95–1.03)	0.57
Proximal neck diameter (mm)	0.92 (0.65–1.30)	0.63
Distal neck diameter (mm)	1.08 (0.82–1.43)	0.57
Neck length (mm)	0.98 (0.92–1.04)	0.46
EL1A at the end of follow-up		
Sex	0.92 (0.86–0.99)	0.03
Age	1.62 (0.53–4.95)	0.39
Infrarenal angulation	0.99 (0.97–1.01)	0.40
Suprarenal angulation	1.00 (0.97–1.03)	0.76
Proximal neck diameter (mm)	1.03 (0.80–1.32)	0.85
Distal neck diameter (mm)	0.86 (0.70–1.06)	0.15
Neck length (mm)	1.04 (0.99–1.09)	0.10

EL1A = endoleak type 1A.

## Data Availability

Data available on request due to restrictions eg privacy or ethical.
